# An Improved Multi-Objective Programming with Augmented *ε*-Constraint Method for Hazardous Waste Location-Routing Problems

**DOI:** 10.3390/ijerph13060548

**Published:** 2016-05-31

**Authors:** Hao Yu, Wei Deng Solvang

**Affiliations:** Department of Industrial Engineering, Faculty of Engineering Science and Technology, UiT—The Arctic University of Norway, Narvik 8505, Norway; wei.d.solvang@uit.no

**Keywords:** hazardous waste management, location-routing problem, mixed integer programming, multi-objective programming, augmented *ε*-constraint method

## Abstract

Hazardous waste location-routing problems are of importance due to the potential risk for nearby residents and the environment. In this paper, an improved mathematical formulation is developed based upon a multi-objective mixed integer programming approach. The model aims at assisting decision makers in selecting locations for different facilities including treatment plants, recycling plants and disposal sites, providing appropriate technologies for hazardous waste treatment, and routing transportation. In the model, two critical factors are taken into account: system operating costs and risk imposed on local residents, and a compensation factor is introduced to the risk objective function in order to account for the fact that the risk level imposed by one type of hazardous waste or treatment technology may significantly vary from that of other types. Besides, the policy instruments for promoting waste recycling are considered, and their influence on the costs and risk of hazardous waste management is also discussed. The model is coded and calculated in Lingo optimization solver, and the augmented *ε*-constraint method is employed to generate the Pareto optimal curve of the multi-objective optimization problem. The trade-off between different objectives is illustrated in the numerical experiment.

## 1. Introduction

During the past few decades, rapid technological development and economic growth have not only improved people’s living standard but also dramatically increased the quantity of hazardous waste generated. Even through more and more non-hazardous materials have been developed to replace hazardous materials in order to achieve sustainable development [1], the amount of hazardous waste generated is still at a high level in both developing and developed economies [2,3,4]. Hazardous waste is characterized as poisonous, carcinogenic, irritant, flammable, reactive, infectious and toxic [5,6,7,8,9], and it can be generated by many industries, *i.e.*, chemical manufacturing process [1], petroleum production [2,6], iron production [6], hospitals [1,7], *etc*. Hazardous waste can have undesirable impacts on the environment directly or indirectly [4]; furthermore, it can also affect people’s health [10], lifestyle [11], and even depreciate the local land value [12]. Proper treatment of hazardous waste is therefore of great importance [6].

Hazardous waste management systems have been planned and established in many countries for dealing with the increased amount of hazardous waste, which involve collection, distribution, treatment, reuse/recycling, and disposal of hazardous waste [6]. The goal of hazardous waste management systems is to collect, transport, treat, recycle and dispose hazardous waste in an economically efficient and environmentally friendly manner [5]. In hazardous waste management system planning, critical decisions have to be made at strategic, tactical and operational levels, among which facility location and transportation route selection are crucial.

The location-routing problem of hazardous waste management has been of great interest to academics and practitioners for some time due to the associated economic and environmental concerns [3,4]. The hazardous waste facility location problem is considered as a “obnoxious” or “undesirable” facility location problem that involves several conflicting objectives [11,13,14], and the optimal solution to one individual objective usually results in undesirable solutions for others [15,16], so the optimal trade-off among all critical influencing factors is the focus of the problem. An early mathematical model was formulated by Koo *et al.* [17], which incorporates integer programming with fuzzy membership functions for determining the location of regional hazardous waste treatment facilities. The model has four objectives and aims at simultaneously balancing risk, equity, public objection, and ease of construction and maintenance. Or and Akgiil [18] propose a mathematical model for minimizing the maximum weighted distance from hazardous waste disposal plants to residential areas in order to decrease the negative impact. Alidi [19] formulates a multi-objective goal programming for the hazardous waste facility location problem accounting for allocated budget, hazardous waste supplies, market selection, number of facilities and facility capacities. Another goal programming for hazardous waste management in the petrochemical industry is given by Alidi [20]. Das *et al.* [21] propose a bi-objective routing model for hazardous waste transportation, and the model uses posteriori multi-objective optimization to generate Pareto frontier between cost and risk. Alcada-Almeida *et al.* [22] and Emek and Kara [7] develop mathematical approaches focusing on the location problem of incineration plants of hazardous waste.

Considering hazardous waste collection, Liu *et al.* [23] propose a scenario-based assessment approach that includes transportation risk, site risk and cost. Four scenarios with different parameters of truckloads, availability of intermediate transfer stations, transportation frequencies, and storage capacities are tested in this study. Li *et al.* [24] develop an integer programming based on Key Player Problem/Positive (KPP-POS) method and covering model for hazardous waste collection problems. The model accounts for two scenarios: the first one aims at determining the maximum coverage of hazardous waste generation points when the number of facilities is fixed (maximal covering), and the other one decides the minimum number of facilities that must be opened in order to cover all the hazardous waste generation points (set covering).

Taking into account both facility location and routing decisions, Giannikos [25] formulates a weighted goal programming for hazardous waste management considering cost, perceived risk, and equitable distribution of risk and disutility. Nema and Gupta [5] propose a bi-objective mixed integer programming for the location-routing problem of hazardous waste management aiming at the balance between cost and risk. The model takes into account the heterogeneous characteristics of different sources of hazardous waste and formulates waste-waste and waste-technology compatibility constraints. Alumur and Kara [1] develop a mathematical model for the hazardous waste location-routing problem, which considers the recycling of hazardous waste including both source recycling and post-treatment recycling. Samanlioglu [6] formulates a multi-objective decision model for the location-routing problem of multi-sourced industrial hazardous waste. The model aims at balancing cost, transportation risk and site risk, and a lexicographic weighted Tchebycheff formulation is proposed to calculate the optimal solutions. Boyer *et al.* [9] propose a mixed integer programming for the location-routing problem of industrial waste. The model considers the system operating costs, transportation risk and site risk of hazardous waste management, and normalized weighted sum method is applied to combine the three objective functions with different measurement units.

Time-varying parameters are the focus in previous mathematical models. Hu *et al.* [26] formulate a multi-period cost minimization model for hazardous waste management. Sheu [27] develops a dynamic reverse logistics model for regional management of industrial hazardous waste, and the model takes into account both cost and risk associated with hazardous waste treatment, transportation and storage. Ardjmand *et al.* [4] propose a bi-objective mathematical programming with stochastic parameters for hazardous material management. Transportation cost is formulated as a stochastic parameter, and a genetic algorithm is also developed for computation in order to improve the computational efficiency of the proposed model. Another genetic approach for the location-routing problem of hazardous waste management is presented by Ardjmand *et al.* [3]. Berglund and Kwon [28] formulate a robust location-routing problem for hazardous waste management. The model monetizes the exposure risk and directly combines it with cost function, and it also formulates both demand uncertainties and transportation risk uncertainties. Furthermore, the independent transportation routing behavior of hazardous waste carriers is also taken into account in this model. Two solution methods are applied in accordance with the size of the problem: the full enumeration method can be used for small problems, while the genetic algorithm approach is applied for medium and large problems.

It is observed from previous mathematical models for hazardous waste management that the topic of most focus is the optimal trade-off between cost and risk, besides, some other objectives, *i.e.*, risk, equity, ease of construction, social opposition, *etc.*, are also incorporated. The models developed in the 1990s are single-sourced waste flow problems where the hazardous waste is considered as homogeneous. However, this is unrealistic because hazardous waste flow is heterogeneous in nature and different technologies are used for dealing with different types of hazardous waste. Due to this, most mathematical models developed in the latest decade are multi-sourced models with the compatibility constraints waste-waste and/or waste-technology. It is also noted the equity minimization objective was formulated in several early researches, but was eliminated in more recent mathematical models for the location-routing problem of hazardous waste because the equity minimization objective usually leads to an increased number of facilities being needed and a reduction of facility utilization.

The objective function of transportation risk and site risk formulated in previous mathematical models is mainly determined by three influencing factors: population exposed, waste amount transported or treated, and probability of accident. However, the connection between risk level and type of hazardous waste, treatment technology applied and local characteristic is not emphasized in previous studies, and this is of paramount importance in decision-making in hazardous waste management system planning, because the consequences may be much more severe when accidents occur at a chemical storage warehouse than at an incineration plant. For example, the explosion of a warehouse for hazardous material storage on 12 August 2015, at Tianjin Port, caused 165 deaths and 698 hospitalizations [29], and 779 businesses suffered losses and more than 17,000 households were evacuated [30]. The overall direct and indirect losses are estimated at more than 31 million US dollars [30], furthermore, the environmental pollution of the soil, surface and ground water, and food supply has a long-term effect on nearby residents’ health [29]. This paper aims at filling the literature gap by developing an improved formulation considering the influence of the compensated risk level determined by the type of waste and treatment technology on hazardous waste management system planning. In addition, the policy instruments for compulsory recycling of the recyclable fraction of post-treatment residue are also a focus of this paper. Policy instruments have been proved to be effective tools for sustainable waste management [31], and some previous models employ one important assumption that all recyclable materials are sent for recycling [1,6,9], however, this may lead to increased system operating costs due to the increase in transportation costs and recycling costs [31,32]. Because of this, this paper also discusses the influence of the compulsory recycling requirement of recyclable fraction of post-treatment residue on hazardous waste management system planning, and it also determines the optimal location-routing plan of each scenario.

In summary, an improved multi-objective mathematical programming approach for the location-routing problem of hazardous waste management is developed in this paper, and the model takes into account the influence of the type of hazardous waste, treatment technology, local characteristics, and policy instruments on decision-making in hazardous waste management system planning. In Section 2, the mathematical model is formulated. In Section 3, the augmented *ε*-constraint method for resolving the multi-objective optimization problem is briefly introduced, and its advantages in *posteriori* decision-making is also discussed. In Section 4, a numerical experiment is given to show the trade-off between different objectives. Section 5 concludes the paper and suggests future work.

## 2. Mathematical Model

The model aims at assisting decision makers in selecting locations of different facilities including treatment plants, recycling plants and disposal sites, equipping appropriate technologies for hazardous waste treatment, and routing transportation. Two objectives are taken into account, namely, minimization of overall system costs and minimization of the risk imposed to nearby residents. The mathematical model developed in this paper is an improved formulation based upon that by Alumur and Kara [1], Samanliogulu [6] and Boyer *et al.* [9], and the main differences and contributions of this model are presented as follows.

1. Our model considers the compensated risk level of different types of hazardous waste to people exposed along the transportation route.
2. Our model considers the compensated risk level of different types of hazardous waste treatment technologies to people exposed around the facility.
3. Our model considers the influence of the requirement of resource recycling on hazardous waste management systems.
4. Our model considers the variable processing costs at treatment facilities, recycling facilities as well as disposal facilities.
5. Our model is resolved by the augmented *ε*-constraint method in order to present a complete Pareto optimal curve for posteriori decision-making.

**Figure 1:**
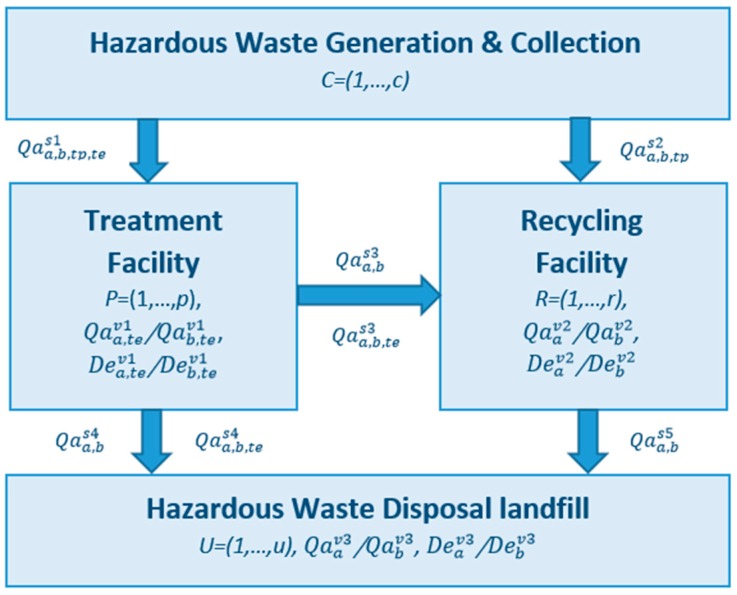
Graphical display of set and decision variables used in the mathematical model.

Figure 1 illustrates the material flow of hazardous waste management systems, and it also presents the sets and decision variables used in the mathematical model, which allows for a better understanding of the model framework [1,6,9]. As shown in the figure, hazardous waste is first collected at generation or collection points and then sent for treatment or recycling. Some types of hazardous waste can be sent for recycling after proper treatment, while the other residues generated at treatment and recycling facilities are sent to landfill. It is noted that hazardous waste cannot be sent to landfill for disposal without proper treatment.

The full list of nomenclatures is given as follows:
**Set:***N* = (*V*, *S*):Set of transportation network formed by nodes *V* and arcs *S**C* = (1, …, *c*):Set of hazardous waste collection points, *C*∈*V**P* = (1, …, *p*):Set of candidate locations of treatment facility, *P*∈*V**R* = (1, …, *r*):Set of candidate locations of recycling facility, *R*∈*V**U* = (1, …, *u*):Set of candidate locations of disposal facility, *U*∈*V**TP* = (1, …, *tp*):Set of types of hazardous waste*TE* = (1, …, *te*):Set of treatment technologies of hazardous waste**Parameters:**$Uc_{a,b}^{s1}$:Unit transportation cost of hazardous waste from collection point *a*∈*C* to treatment facility *b*∈*P*, (*a*, *b*)∈*S*$Uc_{a,b}^{s2}$:Unit transportation cost of hazardous waste from collection point *a*∈*C* to recycling facility *b*∈*R*, (*a*, *b*)∈*S*$Uc_{a,b}^{s3}$:Unit transportation cost of waste components from treatment facility *a*∈*P* to recycling facility *b*∈*R*, (*a*, *b*)∈*S*$Uc_{a,b}^{s4}$:Unit transportation cost of waste residue from treatment facility *a*∈*P* to disposal facility *b*∈*U*, (*a*, *b*)∈*S*$Uc_{a,b}^{s4}$:Unit transportation cost of waste residue from recycling facility *a*∈*R* to disposal facility *b*∈*U*, (*a*, *b*)∈*S*$Fc_{a,te}^{v1}$:Fixed facility cost for opening a treatment facility at candidate point *a*∈*P* with technology *te*∈*TE*$Fc_{a}^{v2}$:Fixed facility cost for opening a recycling facility at candidate point *a*∈*R*$Fc_{a}^{v3}$:Fixed facility cost for opening a disposal facility at candidate point *a*∈*U*$Pc_{a,te}^{v1}$:Unit processing cost at treatment facility *a*∈*P* with technology *te*∈*TE*$Pc_{a}^{v2}$:Unit processing cost at recycling facility *a*∈*R*$Pc_{a}^{v3}$:Unit processing cost at disposal facility *a*∈*U*$RSP_{a,te}$:Risk level of processing technology *te*∈*TE* at treatment facility *a*∈*P*$RSU_{a}$:Risk level of disposal facility *a*∈*U*$RSW_{a,b,tp}$:Risk level of hazardous waste type *tp*∈*TP* transported from collection point *a*∈*C* to treatment facility *b*∈*P*$RSR_{a,b}$:Risk level of post-treatment waste residue transported from treatment facility *a*∈*P* to disposal facility *b*∈*U*$PEP_{a}^{v1}$:Population exposed to treatment facility *a*∈*P*$PEP_{a}^{v3}$:Population exposed to disposal facility *a*∈*U*$PEP_{a,b}^{s1}$:Population exposed along the transportation route from collection point *a*∈*C* to treatment facility *b*∈*P*, (*a*, *b*)∈*S*$PEP_{a,b}^{s4}$:Population exposed along the transportation route from treatment facility *a*∈*P* to disposal facility *b*∈*U*, (*a*, *b*)∈*S*$HWG_{a,tp}$:Hazardous waste type *tp*∈*TP* collected at collection point *a*∈*C*$\tau_{a,te}^{v1}$:Conversion rate of hazardous waste at treatment facility *a*∈*P* with technology *te*∈*TE*$\tau_{a}^{v2}$:Conversion rate of hazardous waste at recycling facility *a*∈*R*$Capt_{a,te}^{v1}$:Capacity of treatment facility *a*∈*P* with technology *te*∈*TE*$Capt_{a}^{v2}$:Capacity of recycling facility *a*∈*R*$Capt_{a}^{v3}$:Capacity of disposal facility *a*∈*U*$ReR_{a,te}$:Required recycling rate of post-treatment residue at treatment facility *a*∈*P* with technology *te*∈*TE*$Fr_{a,te}$:Recyclable fraction of post-treatment residue at treatment facility *a*∈*P* with technology *te*∈*TE*IFN:An infinitive large number$Comp_{tp,\;te}^{v1}$:Binary compatibility factor determines if the type of waste *tp*∈*TP* is compatible with the treatment technology *te*∈*TE*$Comp_{\;tp,b}^{v1}$:Binary compatibility factor determines if the type of waste *tp*∈*TP* is compatible with direct recycling at *b*∈*R*$RS_{x}$:Compensation faction of risk level, $RS_{x}\in \left\{RSP_{a,te},\;RSU_{a},\;RSW_{a,b,tp},\;RSR_{a,b}\right\}$$Compensation_{Consequence}$:Compensation factor of consequence of accident$Compensation_{Probability}$:Compensation factor of probability of accident$Probability_{General}$:General probability of catastrophic accident$Probability_{Locality}$:Locality factor$Consequence_{type}$:Compensation factor for type of hazardous waste$Consequence_{technology}$:Compensation factor for technology applied at treatment facility**Decision variables:**$Qa_{a,b,tp,te}^{s1}$:Amount of hazardous waste type *tp*∈*TP* from collection point *a*∈*C* to treatment facility *b*∈*P* with technology *te*∈*TE*, (*a*, *b*)∈*S*$Qa_{a,b,tp}^{s2}$:Amount of hazardous waste type *tp*∈*TP* from collection point *a*∈*C* to recycling facility *b*∈*R*, (*a*, *b*)∈*S*$Qa_{a,b,te}^{s3}$:Amount of waste components from treatment facility *a*∈*P* with technology *te*∈*TE* to recycling facility *b*∈*R*, (*a*, *b*)∈*S*$Qa_{a,b,te}^{s4}$:Amount of waste residue from treatment facility *a*∈*P* with technology *te*∈*TE* to disposal facility *b*∈*U*, (*a*, *b*)∈*S*$Qa_{a,b}^{s3}$:Amount of waste components from treatment facility *a*∈*P* to recycling facility *b*∈*R*, (*a*, *b*)∈*S*$Qa_{a,b}^{s4}$:Amount of waste residue from treatment facility *a*∈*P* to disposal facility *b*∈*U*, (*a*, *b*)∈*S*$Qa_{a,b}^{s5}$:Amount of waste residue from recycling facility *a*∈*R* to disposal facility *b*∈*U*, (*a*, *b*)∈*S*$Qa_{a,te}^{v1}/Qa_{b,te}^{v1}$:Amount of hazardous waste received at treatment facility *a*∈*P, b*∈*P* with technology *te*∈*TE*$Qa_{a}^{v2}/Qa_{b}^{v2}$:Amount of hazardous waste received at recycling facility *a*∈*R, b*∈*R*$Qa_{a}^{v3}/Qa_{b}^{v3}$:Amount of hazardous waste received at disposal facility *a*∈*U, b*∈*U*$De_{a,te}^{v1}/De_{b,te}^{v1}$*:*Binary decision variable determines if a treatment facility is opened at candidate point *a*∈*P*, *b*∈*P* with technology *tp*∈*TP*$De_{a}^{v2}/De_{b}^{v2}$:Binary decision variable determines if a recycling facility is opened at candidate point *a*∈*R, b*∈*R*$De_{a}^{v3}/De_{b}^{v3}$:Binary decision variable determines if a disposal facility is opened at candidate point *a*∈*U, b*∈*U*


Minimization of overall system operating costs:

(1) $$\text{Mininize Cost}={\text{Cost}}_{\text{fixed facility}}{+\text{Cost}}_{\text{processing}}{+\text{Cost}}_{\text{transportation}}$$

(2) $${\text{Cost}}_{\text{fixed facility}}=\underset{a\in P}{\sum }\underset{te\in TE}{\sum }Fc_{a,te}^{v1}De_{a,te}^{v1}+\underset{a\in R}{\sum }Fc_{a}^{v2}De_{a}^{v2}+\underset{a\in U}{\sum }Fc_{a}^{v3}De_{a}^{v3}$$

(3) $${\text{Cost}}_{\text{processing}}=\underset{a\in P}{\sum }\underset{te\in TE}{\sum }Qa_{a,\;te}^{v1}Pc_{a,te}^{v1}+\underset{a\in R}{\sum }Pc_{a}^{v2}Qa_{a}^{v2}+\underset{a\in U}{\sum }Pc_{a}^{v3}Qa_{a}^{v3}$$

(4) $$\begin{array}{ll} {\mathrm{Cost}}_{\mathrm{transportation}} & =\underset{a\in C}{\sum }\underset{b\in P}{\sum }Uc_{a,b}^{s1}\underset{tp\in TP}{\sum }\underset{te\in TE}{\sum }Qa_{a,b,tp,te}^{s1}+\underset{a\in C}{\sum }\underset{b\in R}{\sum }Uc_{a,b}^{s2}\underset{tp\in TP}{\sum }Qa_{a,b,tp}^{s2}\\ & +\underset{a\in P}{\sum }\underset{b\in P}{\sum }Uc_{a,b}^{s3}Qa_{a,b}^{s3}+\underset{a\in P}{\sum }\underset{b\in U}{\sum }Uc_{a,b}^{s4}Qa_{a,b}^{s4}+\underset{a\in R}{\sum }\underset{b\in U}{\sum }Uc_{a,b}^{s5}Qa_{a,b}^{s5} \end{array}$$

Equation (1) is the objective function of overall system costs, and it includes fixed facility costs, processing costs of hazardous waste, and transportation costs of hazardous waste or residue in each link. Equations (2)–(4) calculate the respective cost components in Equation (1).

Minimization of facility risk:

(5) $$\text{Minimize Risk}=\;{\text{Risk}}_{\text{facility}}{+\text{Risk}}_{\text{transportation}}$$

(6) $${\text{Risk}}_{\text{facility}}=\underset{a\in T}{\sum }PEP_{a}^{v1}\underset{te\in TE}{\sum }Qa_{a,te}^{v1}+\underset{a\in U}{\sum }PEP_{a}^{v3}Qa_{a}^{v3}$$

(7) $${\text{Risk}}_{\text{transportation}}\;=\underset{a\in C}{\sum }\underset{b\in P}{\sum }PEP_{a,b}^{s1}\underset{tp\in TP}{\sum }\underset{te\in TE}{\sum }Qa_{a,b,tp,te}^{s1}+\underset{a\in P}{\sum }\underset{b\in U}{\sum }PEP_{a,b}^{s4}Qa_{a,b}^{s4}$$

Equation (5) is the objective function of risk, and it includes facility risk and transportation risk. Facility risk is directly proportional to the population exposed to the facility and the quantity of hazardous waste processed, as shown in Equation (6). Transportation risk is directly proportional to the quantity of hazardous waste or residue transported and population exposed along the routes, as illustrated in Equation (7). For risk assessment, the most frequently used method can be simplified as: Risk = Consequence × Probability. In Equation (7), the population exposed to the facilities or transportation routes of hazardous waste imply the risk of accident occurring, and the quantity of waste processed or transported is related to the probability of accident, which means the more hazardous waste that is processed and transported, the higher probability of accidents the hazardous waste management system imposes.

Equations (5)–(7) are frequently used in previous mathematical models for quantifying the risk of hazardous waste management, but the different risk levels caused by different treatment technologies and different types of hazardous waste are not considered in this formulation. Facility risk is affected by the risk level of selected technology at the candidate location, and this is of significance because the risk of accident of different technologies for nearby residents is by no means identical. For instance, an explosion of a warehouse of hazardous chemical or radiative materials may lead to much more severe consequences for people and the environment than substandard emissions from an incineration plant. Due to this, Equation (8) is formulated as the compensated risk objective function. Equation (9) calculates the compensated facility risk. In the formula, risk level $RSP_{a,te}$ is determined by treatment technology applied to each candidate location for treatment facility, and risk level $RSU_{a}$ is mainly determined by the candidate locations of landfill. For example, a candidate location with closer proximity to water resources has a larger multiplier of “$RSU_{a}$” due to the potential risk of water pollution caused by leachate leak [33].

Minimization of transportation risk:

(8) $$\text{Minimize Compensated Risk}=\;{\text{ComRisk}}_{\text{facility}}+{\text{ComRisk}}_{\text{transportation}}$$

(9) $${\text{ComRisk}}_{\text{facility}}=\underset{a\in T}{\sum }PEP_{a}^{v1}\underset{te\in TE}{\sum }RSP_{a,te}Qa_{a,te}^{v1}+\underset{a\in U}{\sum }RSU_{a}PEP_{a}^{v3}Qa_{a}^{v3}$$

(10) $${\text{ComRisk}}_{\text{transportation}}\;=\underset{a\in C}{\sum }\underset{b\in P}{\sum }PEP_{a,b}^{s1}\underset{tp\in TP}{\sum }RSW_{a,b,\;tp}\underset{te\in TE}{\sum }Qa_{a,b,tp,te}^{s1}+\underset{a\in P}{\sum }\underset{b\in U}{\sum }RSR_{a,b}PEP_{a,b}^{s4}Qa_{a,b}^{s4}$$

Equation (10) calculates the compensated transportation risk. The risk level “$RSW_{a,b,\;tp}$” is mainly determined by the types of hazardous waste transported from collection points to treatment facilities. For example, the transportation of explosive or radiative hazardous waste may pose much higher risk than the transportation of other types of hazardous waste, so different risk level multipliers are needed for compensating the transportation of different types of hazardous waste. The risk level “$RSR_{a,b}$” of the transportation of residue from treatment facilities to landfill is determined by both types of hazardous waste and selected treatment technology at the candidate location.

The compensation factors of risk level in Equations (9) and (10) are introduced in order to formulate the risk of hazardous waste treatment and transportation in a more appropriate fashion, but the quantification of *RS_x_* is not an easy task. Even through researches have been extensively carried out for risk assessment and risk mitigation of hazardous material transportation [34,35,36], chemical substance storage and processing [37,38,39,40], incineration [41,42] and landfill [42], it is still difficult to find a general framework for risk assessment of hazardous waste management, which enables the quantification of risk level of all types of treatment and transportation on a common basis. In this regard, a simplified method for generalization and quantification of compensation factors of risk level is formulated in Equations (11)–(13).

(11) $$RS_{x}=Compensation_{Consequence}\times Compensation_{Probability}$$

(12) $$Compensation_{Probability}=Probability_{General}\times Probability_{Locality}$$

(13) $$Compensation_{Concsequence}=Consequence_{type}\times Consequence_{technology}$$

The method is formulated based upon the basic risk assessment theory. Equation (11) shows that the compensation factor of risk level *RS_x_* includes two multipliers for compensating both consequence and probability of a catastrophic accident in hazardous waste management. Equation (12) is formulated based on the research conducted by Van Raemdonck *et al.* [36], and it illustrates the compensation factor of probability is determined by the general probability of the accident and locality factor. For example, the locality factor of a candidate location of landfill may be assigned a higher numerical value if the candidate location has a vulnerable geographical structure. Equation (13) measures the consequences of an accident, and it is determined by the type of hazardous waste and technology applied. It is noted that the method is defined in a general form, but not all the compensation multipliers in Equations (11)–(13) can be quantified due to limited information or, in some cases, necessity. For instance, the compensation factor $Consequence_{technology}$ does not need to be considered when the compensation factor of first-level transportation is calculated. Herein, the formulated method mainly aims at presenting a general framework for quantifying the compensation factor of risk level, but due to the complexity of hazardous waste management, the assessment of risk level is usually an ad-hoc process involving a group of experts and all stakeholders.

The constraints of the mathematical model are given in Equations (14)–(32). Equation (14) ensures that each type of hazardous waste generated at each point is sent either for treatment or for recycling.

(14) $$HWG_{a,tp}=\underset{b\in P}{\sum }\underset{te\in TE}{\sum }Qa_{a,b,tp,te}^{s1}+\underset{b\in R}{\sum }Qa_{a,b,tp}^{s2},\;\forall a\in C,\;\forall tp\in TP$$

Constraints (15)–(21) are flow balance requirements for treatment facilities, recycling facilities and disposal facilities, which regulate the relationship between input amount and output amount at respective facilities in the hazardous waste management system.

(15) $$\underset{a\in C}{\sum }\underset{tp\in TP}{\sum }Qa_{a,b,tp,te}^{s1}=Qa_{b,te}^{v1},\;\forall b\in P,\;\forall te\in TE$$

(16) $$\tau_{a,te}^{v1}Qa_{a,te}^{v1}=\underset{b\in R}{\sum }Qa_{a,b,te}^{s3}+\underset{b\in U}{\sum }Qa_{a,b,te}^{s4},\;\forall a\in P,\;\forall te\in TE$$

(17) $$Qa_{a,b}^{s3}=\underset{te\in Te}{\sum }Qa_{a,b,te}^{s3},\;\forall a\in P,\;\forall b\in R$$

(18) $$Qa_{a,b}^{s4}=\underset{te\in Te}{\sum }Qa_{a,b,te}^{s4},\;\forall a\in P,\;\forall b\in U$$

(19) $$\underset{a\in C}{\sum }\underset{tp\in TP}{\sum }Qa_{a,b,tp}^{s2}+\underset{a\in P}{\sum }Qa_{a,b}^{s3}=Qa_{b}^{v2},\;\forall b\in R$$

(20) $$\tau_{a}^{v2}Qa_{a}^{v2}=\underset{b\in U}{\sum }Qa_{a,b}^{s5},\;\forall a\in R$$

(21) $$Qa_{b}^{v3}=\underset{a\in P}{\sum }Qa_{a,b}^{s4}+\underset{a\in R}{\sum }Qa_{a,b}^{s5},\;\forall b\in U$$

Constraints (22)–(24) are capacity restrictions for treatment facilities, recycling facilities and disposal facilities, respectively.

(22) $$Qa_{a,te}^{v1}\leq Capt_{a,te}^{v1}De_{a,te}^{v1},\;\forall a\in P,\;\forall te\in TE$$

(23) $$Qa_{a}^{v2}\leq Capt_{a}^{v2}De_{a}^{v2}\;\forall a\in R$$

(24) $$Qa_{a}^{v3}\leq Capt_{a}^{v3}De_{a}^{v3}\;\forall a\in U$$

Constraints (25) and (26) are compulsory recycling requirements for recyclable fraction of post-treatment residue. Equation (25) ensures the required recycling rate is satisfied at each treatment plant, and Equation (26) specifies the recycled amount of post-treatment residue cannot be more than the recyclable fraction. In previous models [6,9], all the recyclable fraction of post-treatment residue is sent for recycling, but our model, on the other hand, accounts the influence of policy instruments for post-treatment residue on both system operating costs and risk related to transportation and processing; furthermore, the optimal location and routing plan can also be obtained with respect to changing requirements.

(25) $$\frac{\sum_{b\in R}Qa_{a,b}^{s3}}{\sum_{te\in TE}Qa_{a,te}^{v1}\tau_{a,te}^{v1}}\geq \underset{te\in TE}{\sum }ReR_{a,te}De_{a,te}^{v1},\;\forall a\in P$$

(26) $$Fr_{a,te}\geq ReR_{a,te},\;\forall a\in P,\;\forall te\in TE$$

Constraint (27) restricts the maximum number of treatment technologies that can be applied for a selected candidate location. Herein, *InT* is a non-negative integer which specifies how many technologies can be chosen for candidate point a∈P.

(27) $$\underset{te\in TE}{\sum }De_{a,te}^{v1}\leq InT,\;\forall a\in P$$

Constraints (28) and (29) are technological compatibility requirements for treatment facilities and recycling facilities, and they ensure that the hazardous waste can be sent for treatment or recycling only if a candidate location is selected for opening the new facility and the type of hazardous waste is compatible with the technology applied. It is noted that, for direct recycling from the source or collection center, the hazardous waste should be of extremely low transportation risk, e.g., waste electrical and electronic products, but it is not suitable for the hazardous waste with high risk in transportation and processing, e.g., explosive materials.

(28) $$Qa_{a,b,tp,\;te}^{s1}\leq De_{b,te}^{v1}Comp_{\;tp,\;te}^{v1}IFN,\;\forall a\in C,\;\forall b\in P,\;\forall te\in TE,\;\forall tp\in TP$$

(29) $$Qa_{a,b,tp}^{s2}\leq De_{b}^{v2}Comp_{\;tp,b}^{v1}IFN,\;\forall a\in C,\;\forall b\in R,\;\forall tp\in TP$$

Constraints (30)–(32) ensure the transportation of residue between the treatment facility and recycling facility, between the treatment facility and disposal facility, and between the recycling facility and disposal facility may happen only when the respective candidate locations are selected.

(30) $$Qa_{a,b,te}^{s3}\leq De_{a,te}^{v1}De_{b}^{v2}IFN,\;\forall a\in P,\;\forall b\in R,\;\forall te\in TE$$

(31) $$Qa_{a,b,te}^{s4}\leq De_{a,te}^{v1}De_{b}^{v3}IFN,\;\forall a\in P,\;\forall b\in U,\;\forall te\in TE$$

(32) $$Qa_{a,b}^{s5}\leq De_{a}^{v2}De_{b}^{v3}IFN,\;\forall a\in R,\;\forall b\in U$$

In this model, all the decision variables are non-negative variables. Besides, “$De_{a,te}^{v1}$/$De_{b,te}^{v1}$”, “$De_{a}^{v2}$/$De_{b}^{v2}$” and “$De_{a}^{v3}$/$De_{b}^{v3}$” are binary variables which determine whether a new facility will be opened at respective candidate locations, and if the variable equals 1, the candidate point is selected for a new facility, and if the variable equals to 0, otherwise. In addition, the scenario of opening several different types of facilities at one candidate location is not taken into consideration in this model, so sets “*C*”, “*P*”, “*R*”, “*U*” are independent subsets of the set of nodes “*V*” without overlapping elements.

## 3. Solution Method

In previous researches, the most extensively used method for the multi-objective optimization problem of hazardous waste management is the weighted sum method (e.g., Alumur and Kara [1], Nema and Gupta [5], Samanlioglu [6], Boyer *et al.* [9], Rakas *et al.* [11], Yu *et al.* [16], Sheu [27], Yu *et al.* [43]). Equation (33) presents a general form of multi-objective optimization problem (minimum problem), where $g_{k}\left(x\right),\;k=1,\ldots ,z$ represent the objective functions, x is the solution vector of decision variables and Q is the set of feasible solutions. The fundamental principle of weighted sum method is to composite and convert multiple objective functions into a single objective problem through allocating weight to each objective function, as shown in Equation (34). The weight of each objective function $W_{k},\;k=1,\ldots ,z$ implies its relative importance in decision-making. It is noted the measurements of different objectives in a hazardous waste management system are usually not identical, so the objective functions have to be normalized before they can be converted into a single objective optimization problem with the weighted sum method. Equation (35) illustrates an example of a normalized weighted sum method, where $g_{k}^{Min}\left(x\right),\;k=1,\ldots ,z$ is the minimum achievable value of individual objective functions.

(33) $$\text{Min }\theta =\left(g_{1}\left(x\right),g_{2}\left(x\right),\ldots ,g_{z}\left(x\right)\right)\;x\in Q$$

(34) $$\text{Min }\theta =W_{1}g_{1}\left(x\right)+W_{2}g_{2}\left(x\right)+,\ldots ,+W_{z}g_{z}\left(x\right)\;W_{1}+W_{2}+,\ldots ,W_{z}=1\;x\in Q$$

(35) $$\text{Min }\theta =\frac{W_{1}g_{1}\left(x\right)}{g_{1}^{Min}\left(x\right)}+\frac{W_{2}g_{2}\left(x\right)}{g_{2}^{Min}\left(x\right)}+,\ldots ,+\frac{W_{z}g_{z}\left(x\right)}{g_{z}^{Min}\left(x\right)}\;W_{1}+W_{2}+,\ldots ,W_{z}=1\;x\in Q$$

**Figure 2:**
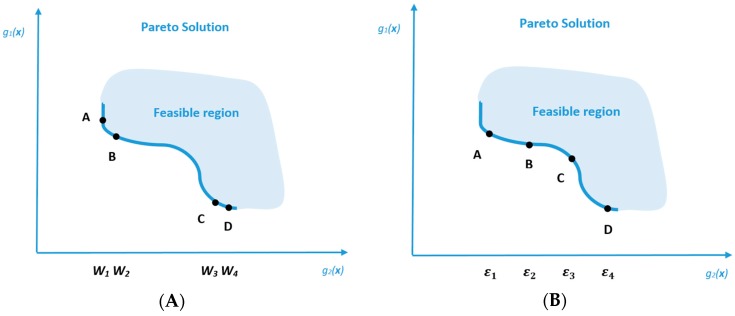
Graphical comparison of the Pareto frontier of a simplified example calculated by: (**A**) Weighted sum method with evenly distributed weight combination; (**B**) *ε*-constraint method with evenly distributed *ε*.

The advantage of the weighted sum method is simplicity, because the method can be easily used to determine the trade-off between different objectives with respect to the given weights. Besides, the weighted sum method also enables the interaction between the subjective preference of decision makers and objective information of the system by varying the weight combinations of different objective functions. However, on the other hand, the weighted sum method has some weaknesses in resolving multi-objective optimization problems [44]. Weighted sum is an *a priori* method, which means the weight of each objective function must be pre-determined and the optimal result obtained is significantly affected by the given weights. Therefore, the weighted sum is not an effective method when the relative importance of each objective is unclear or cannot be pre-determined by decision makers, and which is frequently encountered in the system planning of hazardous waste management. For *posteriori* decision-making, the weighted sum is neither able to generate evenly distributed Pareto solutions nor a complete set of points at the Pareto frontier. A simplified example is given in Figure 2, which illustrates the inability of the weighted sum method to determine the Pareto frontier for a non-convex feasible region, and more details on the weaknesses of the weighted sum method are given by Das and Dennis [44].

*Posteriori* methods are used to generate an evenly distributed and complete Pareto frontier, among which the ε-constraint method is the most extensively used one. A general form of ε-constraint method is formulated in Equation (36). As shown in the formula, the multi-objective optimization problem becomes a single objective optimization problem by converting the other objective functions into constraints with the help of ε, and, in this way, it is not necessary to normalize the objective functions even if they are measured by different units. Compared with the weighted sum method, the performance of ε. *ε*-constraint method in generating the Pareto frontier is much better as illustrated in Figure 2.

(36) $$\text{Min }g_{1}\left(x\right)\;g_{2}\left(x\right)\leq \varepsilon_{2}\;g_{3}\left(x\right)\leq \varepsilon_{3}\;\ldots \;g_{z}\left(x\right)\leq \varepsilon_{z}\;x\in Q$$

The value selected for *ε* is determined by payoff matrix, and it has great influence on the Pareto frontier generated. The payoff matrix calculated by conventional *ε*-constraint method may lead to dominated or weakly efficient solutions which result in an unevenly distributed Pareto optimal curve [45]. In order to solve this problem, improved *ε*-constraint methods have been developed during the past decade [45,46,47]. In this paper, the augmented *ε*-constraint method introduced in Mavrotas [45] is employed to generate the Pareto solutions of the multi-objective optimization problem of hazardous waste management. 

**Figure 3:**
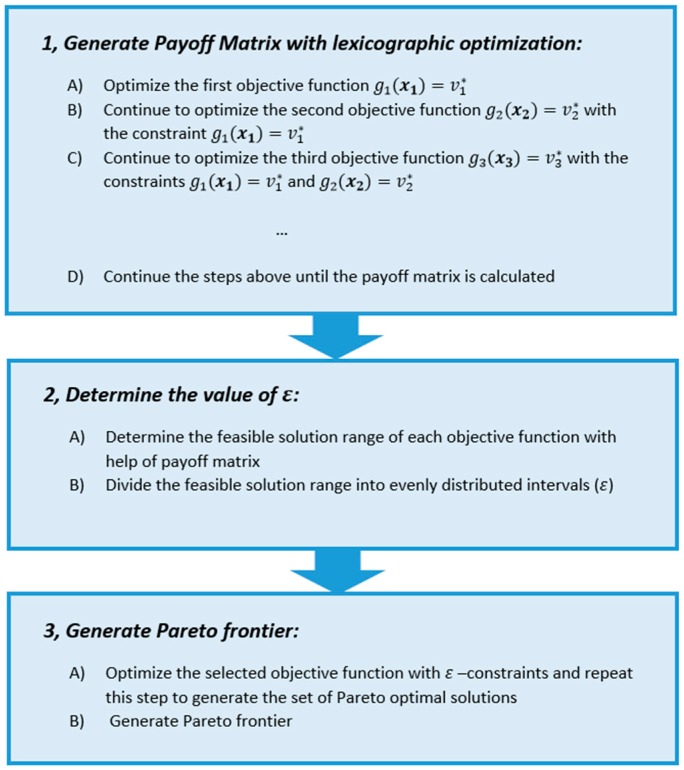
Solution procedures of augmented *ε*-constraint method.

Figure 3 presents the procedures to generate the Pareto optimal curve using the augmented *ε*-constraint method. As shown in the figure, the lexicographical optimization is used continually to optimize a series of objectives in calculating the payoff matrix. This method can effectively eliminate the dominated optimal solutions in the payoff matrix, which are usually encountered with conventional calculations of the payoff matrix, and the evenly distributed Pareto optimal curve is therefore guaranteed with this method.

## 4. Numerical Experiment and Discussion

In this section, a numerical experiment is performed in order to demonstrate the applicability of the model and to present some managerial insights on hazardous waste management system design. The example includes twenty collection points for hazardous waste, eight candidate locations for treatment facilities, six candidate locations for recycling facilities, six candidate locations for landfill and four types of hazardous waste (A, B, C and D). We assume two types of treatment technology can be applied: incineration and chemical treatment. The technological compatibility logic for different types of hazardous waste is given as follows.

- Type A is suitable for direct recycling.
- Type B can only be treated by incineration.
- Type C can only be treated by chemical treatment.
- Type D can be treated either by incineration or by chemical treatment.

In this example, the test parameters are randomly generated by giving an interval. For example, the population of each hazardous collection point is randomly generated between 30,000 and 100,000, and, in this case, they are 58,042, 64,891, 70,914, 74,466, 51,911, 49,956, 32,487, 95,449, 77,903, 43,743, 32,885, 36,731, 36,645, 70,031, 89,961, 62,111, 82,042, 36,559, 61,617 and 53,537, respectively. The generation of hazardous waste is directly proportional to the population, so a multiplier randomly generated between 0.0015 and 0.003 ton/year per capita is introduced to convert the population into the amount of different types of hazardous waste generated at each collection point. The other test parameters are generated in the same way, and the intervals used for parameter generation for the numerical experiment are given in Appendix A (Table A1 and Table A2).

The parameter intervals are given based upon the relevant information used in previous literature [1,6] in order to maintain the rationality of the example. It is noted that the conversion rate and recyclable fraction are inversely proportional to the processing cost, and it means better performance usually increases the processing costs at treatment facilities. For example, the use of better catalysts or combustion promoters can improve the chemical reaction or incineration process and then result in a higher conversion rate and recyclable fraction of the residue, but it also increases the cost for processing the hazardous waste. In addition, the unit transportation cost of hazardous waste is higher than the unit transportation cost of residue, because transportation of hazardous waste requires more care and specialized equipment than does the residue [6]. The recyclable fraction of the post-treatment residue is relatively small due to the hazardous substances it contains [1,6].

**Figure 4:**
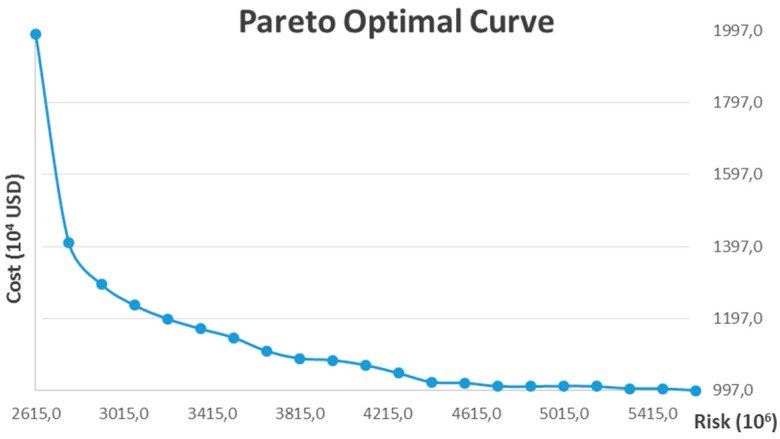
Pareto optimal curve of the example: Cost *vs.* Risk.

In the first scenario, the compulsory recycling requirement for the recyclable fraction of post-treatment residue is not incorporated, which means Constraints (25) and (26) are relaxed. Furthermore, we assume all the candidate locations for treatment facility can simultaneously allow for the opening of both an incineration plant and a chemical treatment plant, which means *InT* equals 2. The model is coded with a Lingo optimization solver and resolved on a personal computer with 4 GB RAM. The payoff matrix is first calculated by a lexicographical method and is shown in Table 1. The Pareto optimal curve is then generated in Figure 4, which provides a set of “optimal combinations” to decision makers. The model includes 1956 decision variables among which 28 are integer variables, so it is considered as a medium or large sized problem. The CPU time required for computing the global optimum of different scenarios with increasing value of *ε* varies significantly, and, in most scenarios, the global optimum can be calculated in 2–30 min. However, the CPU time increases dramatically when the optimal individual cost is approached, and computation of the global optimum of some scenarios requires up to 8 h.

As shown in Figure 4, the Pareto optimal curve shows a convex property and explicitly presents the trade-off between system operating costs and risk to nearby residents. In general, more has to be spent in order to reduce the risk of the processing and transportation of hazardous waste. Besides, it is also observed that the slope of the Pareto optimal curve increases significantly with the decrease of risk. This reflects the economic efficiency of risk mitigation for hazardous waste management, say, if we slightly increase the minimum individual cost, the risk can be significantly reduced, however, the economic efficiency of risk mitigation gradually decreases with the increase of investment.

(37) $${\text{Economic Efficiency of risk mitigation}}_{\left(x,\;x\prime \right)}=\frac{Risk_{x}-Risk_{x\prime }}{Cost_{x\prime }-Cost_{x}}$$

Equation (37) is formulated to calculate the economic efficiency of risk mitigation, which quantifies how much risk can be reduced by a unit cost, and this provides decision makers with valuable information and deep insights for the design of hazardous waste management systems. For example, it can be suggested to decision makers that the risk of hazardous waste management systems can be reduced by approximately 42.7% by increasing the investment by 10% from the individual cost optimization scenario, and the economic efficiency of risk mitigation is 4.27 (10^6^ risk/10^4^ USD) in this stage. However, if we continue to increase the investment by another 20%, the risk can only be reduced by approximately 8%, and the economic efficiency of risk mitigation becomes 0.4 (10^6^ risk/10^4^ USD) in this stage. Further, when the investment continues to increase to more than 40% from minimum individual cost, the economic efficiency becomes much lower, and this implies much more has to be spent in this stage to reduce risk. The results given by the example have great influence on decision-making in hazardous waste management system design and planning.

**Figure 5:**
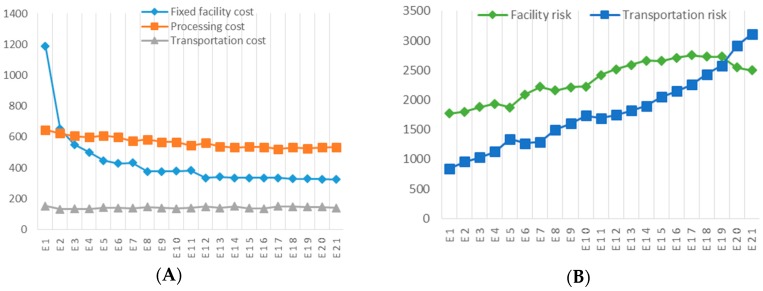
Cost components and risk components over Pareto optimal curve: (**A**) Comparison of fixed facility cost, processing cost and transportation cost (10^4^ USD); (**B**) Comparison of facility risk and transportation risk (10^6^).

Figure 5 illustrates the cost components (A) and risk components (B) over the Pareto optimal curve. As shown in the figure, the fixed facility cost changes in a similar way to the change in overall system operating costs, and with the increased risk of hazardous waste management, the variable processing cost gradually decreases while the transportation cost remains relatively stable. Over the Pareto optimal curve, the transportation risk increases more rapidly compared with the increase of facility risk, particularly when the maximum risk scenario is approached. This implies facility selection and operation have more significant influence on system operating costs while transportation plays a more important role in determining the risk of the hazardous waste management system.

Compared with previous studies which can only provide one optimal solution with a pre-determined combination of weights (e.g., Alumur and Kara [1], Emek and Kara [7], Yu *et al.* [16] and Sheu [27]) or several optimal solutions with changing combinations of weights (e.g., Samanlioglu [6] and Omid *et al.* [9]), the result calculated by augmented *ε*-constraint method can present a complete set of non-dominated optimal solutions. This means, with the help of augmented *ε*-constraint method, our focus is not to determine the optimal network configuration (facility location, technology selection and routing transportation) of hazardous waste management system with respect to the pre-determined subjective importance of different objectives, but it is to analyze the trade-off between the cost and risk of hazardous waste management in an objective fashion. The advantage is that decision makers do not need to make a “rush” decision on the relative importance of different objectives without conducting an overall analysis of the trade-off between cost and risk, and this enables more rational and effective decision-making in the design and planning of hazardous waste management systems. However, as we always see, there are “two sides to the same coin”, one positive side and one negative side. The augmented *ε*-constraint method requires much more CPU time for calculating the Pareto optimal curve due to the fact that several rounds of calculation have to be performed in order to identify non-dominated solutions located at the Pareto frontier.

In this example, we are interested in the influence of the compulsory recycling requirement of recyclable fraction of post-treatment residue on the performance of the hazardous waste management system. Hence, sensitivity analysis is conducted with different compulsory recycling requirements: (A) Scenario-1: no requirement; (B) Secenario-2: 50% of the recyclable fraction of post-treatment residue has to be recycled; (C) Scenario-3: 100% of the recyclable fraction of post-treatment residue has to be recycled.

**Figure 6:**
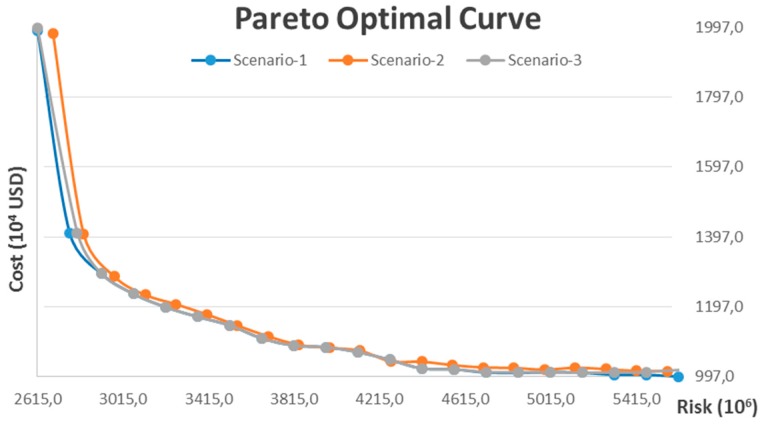
Comparison of Pareto optimal curves in different scenarios.

**Figure 7:**
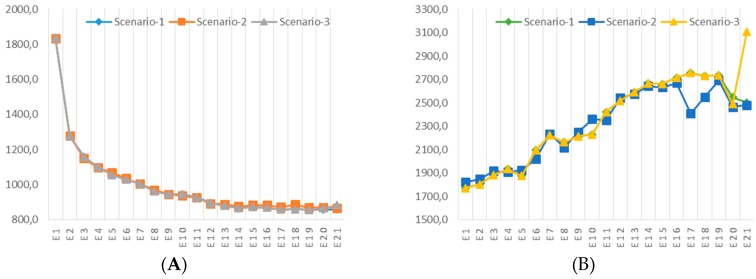

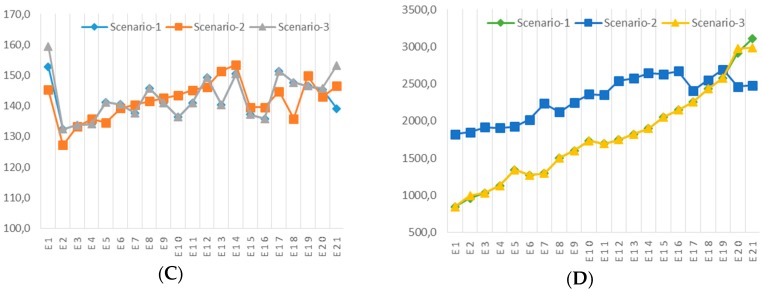
Comparison of cost components and risk components in different scenarios: (**A**) Comparison of facility cost (10^4^ USD); (**B**) Comparison of facility risk (10^6^); (**C**) Comparison of transportation cost (10^4^ USD); (**D**) Comparison of transportation risk (10^6^).

The results of sensitivity analysis are given in Figure 6 and Figure 7. Figure 6 illustrates the comparison between Pareto optimal curves in different scenarios. As shown in the figure, the varying requirements will not greatly change the trade-off between system operating costs and risk of hazardous waste management. The most significant difference arises when only the cost minimization objective is accounted for, which means a more stringent compulsory recycling requirement for the recyclable fraction of post-treatment residue results in a higher individual minimum cost. However, when risk mitigation is taken into account, the Pareto optimal curves of scenario-1 and scenario-3 overlap with each other until the individual minimum risk is approached, and the Pareto optimal curve of scenario-2 is slightly higher. This means recycling 100% of the recyclable fraction of post-treatment residue is an appropriate choice for risk mitigation of the hazardous waste management system regardless of whether the compulsory recycling requirement is implemented, and the result also suggests to decision makers that implementing a compulsory recycling requirement of 100% recyclable post-treatment residue allows for better economic efficiency, as per this example.

Figure 7 presents the comparison of facility cost, transportation cost, facility risk and transportation risk of different scenarios. This provides decision makers with deep insight into how compulsory recycling requirements affect different cost elements and risk elements in hazardous waste management.

## 5. Conclusions

In this paper, we introduce an improved formulation with an augmented *ε*-constraint method for the location-routing problem of hazardous waste management. The most important contribution is that, first, we consider the risk level of different types of hazardous waste and different treatment technologies, through which the location-routing problem of hazardous waste management can be represented and formulated in a more realistic manner. When the risk level of different types of hazardous waste and different treatment technologies is not accounted for, the risk level for a warehouse of radiative materials and an incinerator at the same location is evaluated as being the same. However, the consequences from an accident occurring at these two types of facilities may vary significantly, and the improved formulation can effectively differentiate between risk levels and therefore provides a more appropriate risk assessment for hazardous waste management. Second, we include the variable processing costs for hazardous waste treatment, which influence both system operating costs and selection of treatment technology. Third, we use the augmented *ε*-constraint method to generate the Pareto optimal curve for *posteriori* decision making, and to our knowledge, it is the first attempt to calculate and present a complete Pareto frontier for the location-routing problem of hazardous waste management. Compared with the solution methods used in previous studies, the augmented *ε*-constraint method can not only determine the optimal network configuration of hazardous waste management systems with respect to one or several given scenarios but also provides a complete combination of optimal scenarios. Fourth, we formulate and discuss the influence of compulsory recycling requirements for the recyclable fraction of post-treatment residue on hazardous waste management, and this provides decision makers with valuable information, particularly when a new policy instrument is discussed or implemented.

The mathematical model is formulated as a multi-objective mixed integer programming, and it is coded and resolved with Lingo optimization solver. A numerical experiment is performed to show the applicability of the model and to provide extensive managerial insight, as well. In the numerical example, the Pareto optimal curve of the given problem is first generated, which shows the trade-off between system operating costs and risk of hazardous waste management. It also presents the economic efficiency of risk mitigation, which provides valuable information in decision making. The result reveals that a small increase in investment can drastically reduce the risk imposed to local residents, but the cost effectiveness in risk mitigation decreases rapidly with the continuous increase of investment. Sensitivity analysis with two different compulsory recycling requirements of post-treatment residue is then conducted, and the result provides decision makers with knowledge about the effectiveness of policy instruments in hazardous waste management. In this example, recycling of 100% of the recyclable fraction of post-treatment residue is proven to be the most effective policy.

This paper aims to present a novel idea in formulating the location-routing problem of hazardous waste management considering the compensation of risk objective and, applying the augmented *ε*-constraint method, it also allows for a more comprehensive understanding of the trade-off between system operating costs and the risk associated with hazardous waste management. For further development, three research directions are suggested.

First, this paper presents the idea to incorporate the risk objective function with the compensation factor to design more effective hazardous waste management systems. However, quantifying the risk multiplier on a common basis is not an easy task, and it usually requires the involvement of a large number of experts and stakeholders. Due to this, the development of a more comprehensive framework for risk assessment of the processing and transportation of hazardous waste is first suggested in order to measure and quantify different risks on a common basis.

Second, hazardous waste management involves many uncertainties. For example, the generation of different types of waste may have great seasonality, the transportation costs may be significantly influenced by the fuel price which usually fluctuates, and so forth. Hence, the model can be further developed in a stochastic environment for decision-making with uncertainties.

Last but not least, the location-routing problem of hazardous waste management combines two NP-hard problems: vehicle routing and facility location [48,49], so it is also a NP-hard problem [6]. With the increase of the size of the problem, it will eventually become computationally unmanageable, as illustrated by Emek and Kara [7]. Due to this, some advanced algorithms for location-routing problem have been developed [50,51,52], but the application in hazardous waste management is scarce. To our knowledge, only two examples can be found in Ardjmand [3] and Ardjmand [4]. Therefore, we suggest the use of more advanced computational algorithms in future studies for resolving the location-routing problem of hazardous waste management.

## Figures and Tables

**Table 1:** Payoff matrix calculated by lexicographical method.

	Cost (10^3^ USD)	Risk (10^6^)
Min Cost	9972	5612
Min Risk	19,887	2615

## Appendix A: Generation of Test Parameters and Technological Compatibility Matrix

**Table A1:** Random intervals for parameters.

Parameter	Interval
Population of hazardous waste collection points	(30,000, 100,000)
Generation of each type of hazardous waste at each collection point	(0.0015, 0.003) × Population (ton/year)
Fixed cost of treatment facility (incineration and chemical treatment), recycling facility and disposal facility	(500, 700); (400, 550); (200, 300); (200, 300) (10^3^ USD/year)
Processing cost at treatment facility (incineration and chemical treatment), recycling facility and disposal facility	(500, 800); (400, 500); (300, 500); (100, 200) (USD/ton)
Capacity of treatment facility (incineration and chemical treatment), recycling facility and disposal facility	1500; 2500; 2500; 2500 (ton)
Recyclable fraction at treatment facility (incineration and chemical treatment)	Conversion Multiplier × (1/processing cost) (100%)
Conversion rate at treatment facility (incineration and chemical treatment) and recycling facility	70%; 40%; 50%
Population exposed to treatment facility (incineration and chemical treatment) and disposal facility	(2000, 10,000); (500, 1000)
Risk level of treatment facility (incineration and chemical treatment) and disposal facility	Conversion Multiplier × (1/processing cost)
Proximity between different nodes	(10, 50) (kilometer)
Unit transportation cost	(4, 8) × Proximity for link *s1* and *s2*; (2, 5) × Proximity for the others (USD/ton)
Population along the route between collection point and treatment facility, and between treatment facility and disposal facility	(100, 300) × Proximity
Risk level of different type of hazardous waste transported between collection point and treatment facility	(2, 5) × Proximity
Risk level of the residue transported between treatment facility to disposal facility	(1, 3) × Proximity

**Table A2:** Compatibility of waste type-treatment technology.

	Recycling	Incineration	Chemical Treatment
Type A	√		
Type B		√	
Type C			√
Type D		√	√
